# *splicekit*: an integrative toolkit for splicing analysis from short-read RNA-seq

**DOI:** 10.1093/bioadv/vbae121

**Published:** 2024-08-17

**Authors:** Gregor Rot, Arne Wehling, Roland Schmucki, Nikolaos Berntenis, Jitao David Zhang, Martin Ebeling

**Affiliations:** Roche Pharmaceutical Research and Early Development, Roche Innovation Center Basel, Basel, Switzerland; Roche Pharmaceutical Research and Early Development, Roche Innovation Center Basel, Basel, Switzerland; Roche Pharmaceutical Research and Early Development, Roche Innovation Center Basel, Basel, Switzerland; Roche Pharmaceutical Research and Early Development, Roche Innovation Center Basel, Basel, Switzerland; Roche Pharmaceutical Research and Early Development, Roche Innovation Center Basel, Basel, Switzerland; Roche Pharmaceutical Research and Early Development, Roche Innovation Center Basel, Basel, Switzerland

## Abstract

**Motivation:**

Analysis of alternative splicing using short-read RNA-seq data is a complex process that involves several steps: alignment of reads to the reference genome, identification of alternatively spliced features, motif discovery, analysis of RNA-protein binding near donor and acceptor splice sites, and exploratory data visualization. To the best of our knowledge, there is currently no integrative open-source software dedicated to this task.

**Results:**

Here, we introduce *splicekit*, a Python package that provides and integrates a set of existing and novel splicing analysis tools for conducting splicing analysis.

**Availability and implementation:**

The software *splicekit* is open-source and available at Github (https://github.com/bedapub/splicekit) and *via* the Python Package Index.

## 1 Introduction

Alternative splicing of RNA is a fundamental biological process that is critical for the generation of protein diversity. Dysregulation of splicing has been implicated in many human diseases such as cancer and neurological disorders (Scotti and Swanson 2016). Recent advances in splicing modulation using compounds, i.e. small molecules (Schneider-Poetsch *et al.* 2021), such as Risdiplam for the treatment of spinal muscular atrophy (Ratni *et al.* 2018), have renewed interest in developing new therapies targeting splicing.

Splicing analysis using short-read RNA-seq data is a multifaceted process that involves several steps and requires the integration of diverse software tools. Typically, only a small fraction of sequencing reads are informative for analysis of splicing events. Once such events have been identified, hypotheses about underlying mechanisms often rely on analysis of splice site patterns, or the presence of regulatory elements proximal to a differentially spliced event.

Current tools addressing comprehensive analysis of splicing (e.g. ASTK, Huang *et al.* 2024; SpliceTools, Flemington *et al.* 2023) offer a vast range of possibilities at different levels of complexity. Our approach was to envision an easy-to-use tool that would allow fast analysis of several datasets, with emphasis on connectivity to existing tools and requirements such as explorative visualizations, generation of RNA-maps, and discovery of splice site regulation *de novo* (without the help of existing exon annotations).

**Figure 1.**
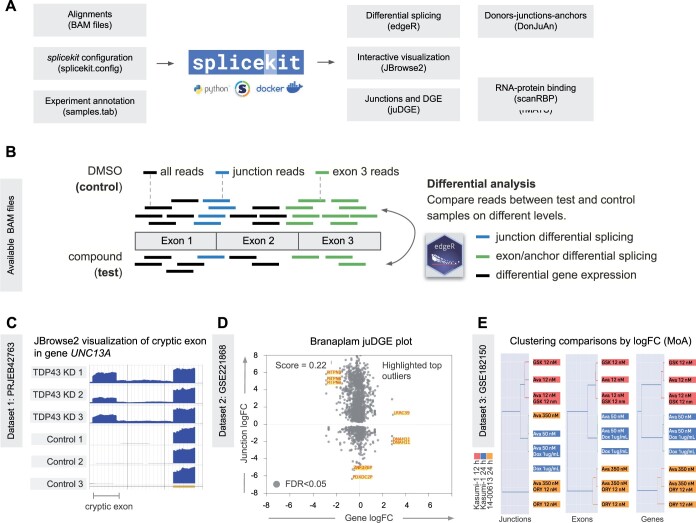
splicekit: an integrative toolkit for splicing analysis from short-read RNA-seq. (A) Overview: input to *splicekit* is alignments of short RNA-seq reads to reference genome (BAM files) together with a sample annotation and *splicekit* configuration file. Results are produced from several *splicekit* modules: differential splicing analysis (edgeR), data visualization with JBrowse2, splicing event analysis with rMATS, RNA–protein binding analysis with scanRBP, and DonJuAn with motif analysis using DREME. (B) Initial input to *splicekit* is read alignments in BAM format. Comparisons are made between groups of test and control experiments. After initial differential calling on the level of splicing (junctions, exons) and genes, further downstream analysis includes JBrowse2 visualizations, juDGE plots [log2 fold change (logFC) of genes and junctions], motif, and RNA-binding protein enrichment analysis. (C) JBrowse2 visualization of PRJEB42763 samples and UNC13A cryptic exon. The cryptic exon is reported by *splicekit* junction analysis. (D) Junction logFC versus gene expression logFC (juDGE) plot in GSE221868 suggest Branaplam is a splicing modifying compound. A low juDGE score (stdev_x/stdev_y) suggests that most regulation happens at the splicing (junction) level. (E) Clustering of comparisons (test conditions versus controls) at the junction, exon, and gene levels. Only features with FDR < 0.05 in at least one comparison.

To address this need, we introduce *splicekit*, a Python package that provides and integrates a set of existing and novel splicing analysis tools (Fig. 1A). It offers functionalities to identify differentially expressed features (junctions, exons, and genes), cluster samples, perform motif analysis to elucidate potential regulatory patterns, visualize changes of junctions versus those of genes, and to identify RNA–protein binding and regulatory motifs in the vicinity of regulated features.

For the integrated analysis of differential splicing events, the community can potentially benefit from a comprehensive and efficient analysis toolbox which is convenient to use, flexible, and modular.

## 2 Methods

### 2.1 Differential splicing

The first step in *splicekit* is to identify regulated features in the comparison, for which splicekit runs edgeR (Robinson *et al.* 2009, Chen *et al.* 2016, Chen *et al.* 2024) with the diffSpliceDGE function to estimate differential splicing (on junction and exon counts within their respective gene context) and the edgeR glmQLFTest function to estimate differential gene expression.

### 2.2 Efficient genome management with pybio

Internally, *splicekit* relies on the Python package pybio that we created to streamline the management of Ensembl and other genome assemblies and annotations. This package offers an interface to downloading and processing of existing Ensembl genomes, while also offering the capability to input a custom genome assembly (FASTA) and annotation (GTF). A key feature of pybio is its rapid indexing of genome annotations, which enables efficient search of features such as exons and genes by genomic position, as well as fast sequence retrieval from provided genomic coordinates.

### 2.3 JBrowse2 visualization

To visualize read coverage and alignments, *splicekit* provides an integrated JBrowse2 instance (Diesh *et al.* 2023) using a local Python web server. Results reported by *splicekit* are decorated with URL links to JBrowse2. For instance, when examining differentially regulated exons or junctions, the URLs point to the region encompassing the feature and automatically select coverage plots for the relevant control and test samples (Fig. 1C).

### 2.4 Junction-DGE and cluster logFC plots

To determine whether treated samples exhibit predominantly splicing or gene expression changes compared to control samples, *splicekit* generates junction-DGE (juDGE) plots (Fig. 1D). Each point in a juDGE plot represents a gene junction. The *y*-axis plots log2 fold changes (logFC) of the junctions, while the *x*-axis plots logFC of genes, resulting in junctions from the same gene having the same *x* coordinate in the plot. By incorporating both genes and junctions and plotting gene logFC against junction logFC, we can estimate the extent of alternative splicing relative to differential gene expression. A tall vertical plot with a low plot score, defined by the ratio of standard variance of *x*- and *y*-axis values, indicates that the detected changes are primarily at the splicing level. In contrast, a wider horizontal plot (high score) suggests significant differential gene expression changes.

For multiple comparisons, *splicekit* also offers logFC clustering analysis at the junction, exon, and gene levels (Fig. 1E).

### 2.5 RNA–protein binding analysis with scanRBP

To explore the possible participation of RNA–binding proteins (RBPs) in the mechanisms of observed differential splicing events, we developed an RBP analysis tool called scanRBP. This tool generates cumulative CLIP data plots (König *et al.* 2010, van Nostrand *et al.* 2016) surrounding a group of regulated features or employs mCrossAtlas and its 112 position weight matrices (PWMs) derived from eCLIP data (Feng *et al.* 2019) to visualize log-odds signals across provided sequences. We additionally extended scanRBP to include PWMs from another database (202 PWMs from CISBP-RNA, Ray *et al.* 2013) displaying the capability of scanRBP to compute predictive binding from PWMs of diverse proteins and experimental designs and experimental binding directly from analyzed CLIP experiments. The resulting RNA-maps can aid in suggesting potential roles of RBPs in differential splicing (Rot *et al.* 2017).

**Figure 2.**
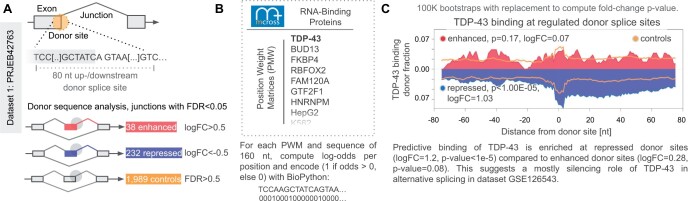
scanRBP analysis. (A) Identification of regulated junctions (FDR < 0.05) performed with edgeR (with gene context), and then classification of donor splice sites to enhanced (logFC > 0.5), repressed (logFC < −0.05), and controls (FDR > 0.5). Sets of sequences for the classes are generated by taking the [−80.80] nt region with the donor splice site at the center. (B) Taking PWMs from mCrossBase, scanRBP can compute log-odds signals (binding vectors) of 112 diverse RBPs across provided sequences. Only signals >0 are considered and binarized (if signal >0 = 1, otherwise 0). (C) scanRBP predictive binding (PWM on sequence) analysis of TDP-43 RNA–protein binding in PRJEB42763 suggests enrichment of TDP-43 binding at repressed sites. The experimental binding eCLIP plot generated using ENCODE dataset ENCSR720BJU (K562 cells) suggests a similar repressive trend and is shown in Supplementary Fig. S4.

Additionally, we compared the scanRBP RNA-maps generated using predictive binding (Fig. 2C) and actual binding using eCLIP (Supplementary Fig. S4). In the TDP-43 context, the analysis suggests predictive binding using PWMs is of value however caution in the interpretation of results is crucial.

### 2.6 Donor-junction-anchor

**Figure 3.**
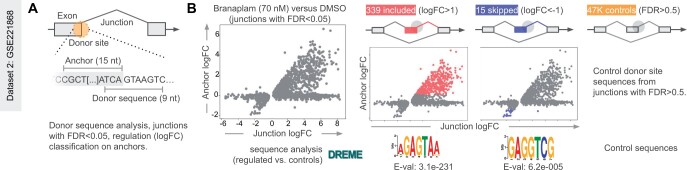
Donor-junction-anchor (DonJuAn). (A) Identification of significantly regulated donor splice sites with DonJuAn. First, we detect significantly changed junctions (FDR < 0.05). We then construct anchors (15 nt regions upstream the donor splice site). The classification of donor sites is performed by looking at significant junctions and logFC of anchors, resulting in sets of included (logFC > 1), skipped (logFC < −1), and control donor sites (FDR > 0.5). (B) Donor-junction-anchor (DonJuAn) on Branaplam versus DMSO in GSE221868 identifies relevant donor sites to detect known binding motif AGAGTAA (Palacino *et al.* 2015).

Small molecular splicing modifiers are an arising therapeutic modality that targets exon inclusion rates (Sivaramakrishnan *et al.* 2017). To identify sequence specific splicing effects, we implemented the DonJuAn (donor-junction-anchor) module in *splicekit*. DonJuAn identifies the donor site sequences and exonic anchor regions of the detected junctions for differential expression analysis. Exon inclusion produces positive junction/anchor logFC values, while exon skipping events give negative values which allows for filtering (Fig. 3A). As a demonstration, we analyzed public data (Ishigami *et al.* 2024) on Branaplam, an experimental drug to treat spinal muscular atrophy that binds to donor splice sites, surrounded by the sequence GAGTAAGT (Palacino *et al.* 2015). DonJuAn logFC junction stratification (Fig. 3B) increases the signal for motif enrichment software such as DREME (Bailey 2011).

### 2.7 Splicing event level analysis with rMATS

*splicekit* does not categorize identified splicing changes at the junction and exon levels into specific splicing events (such as exon skipping, alternative 5′/3′ splice sites, intron retention, or mutually exclusive exons). For those interested in examining splicing at the event level, we offer an interface to rMATS (Shen *et al.* 2014) analysis, which can be executed automatically through *splicekit*.

### 2.8 Interactive report

**Figure 4.**
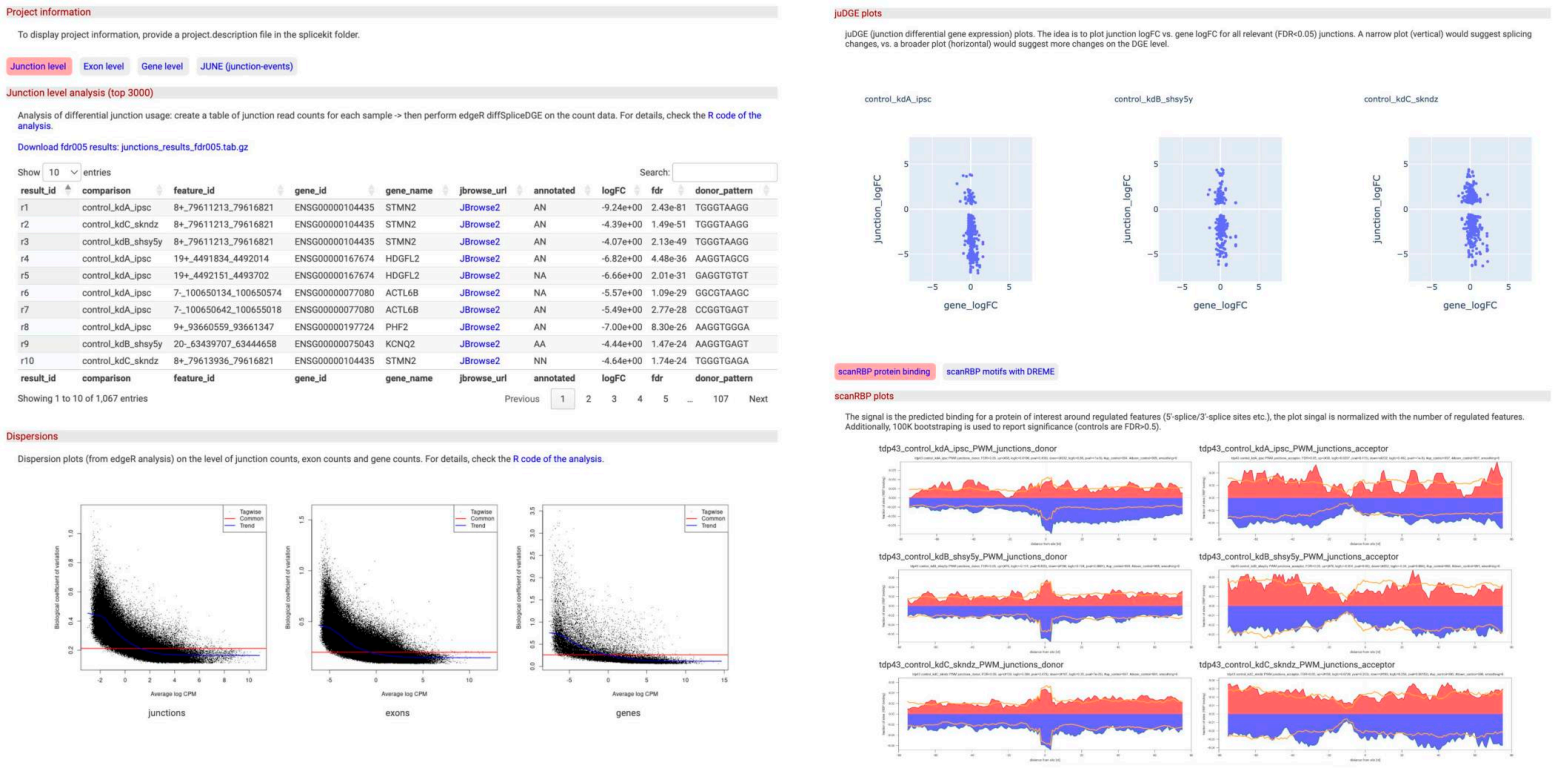
Interactive report. An interactive html report is generated (partially shown) that includes searchable regulated features (at the junction, exon, and gene levels) with direct links to JBrowse coverage web-pages, dispersion plots, juDGE, and scanRBP plots together with motif analysis.

An interactive HTML report is produced (Fig. 4), offering a user-friendly interface for navigating and querying a comprehensive array of analysis results. This includes detailed assessments of feature differential usage at the junction, exon, and gene levels, as well as insightful juDGE plots, scanRBP plots, and motif analysis. The streamlined and intuitive search functionality embedded within the report provides a panoramic view of the entire *splicekit* analysis, facilitating a quick yet thorough examination of the findings.

### 2.9 Usability and containerization

As the extensive integrative analysis, which involves calling multiple scripts and tools, can be difficult to install due to numerous software dependencies, we simplify this process using Docker containerization. It is straightforward to run *splicekit* by installing the Python package with pip install *splicekit* and running the container with docker or singularity. This will import the online Docker image from GitHub. The container supplies all the necessary software.

## 3 Results

To demonstrate the comprehensive and extensive analytical capabilities of *splicekit*, we conducted analyses on multiple publicly accessible RNA-seq datasets.

### 3.1 Characterization of differential splicing in a cellular model of TDP-43 knockout

TDP-43, or TAR DNA-binding protein 43, plays a crucial role in the regulation of alternative splicing, including the suppression of non-conserved cryptic exons. Its dysfunction is associated with abnormal splicing events that can contribute to neurodegenerative diseases such as amyotrophic lateral sclerosis (ALS) and frontotemporal dementia (FTD).

Since the basis of *splicekit* analysis is the identification of regulated features (Fig. 1B), we were able to immediately identify the cryptic exon in gene UNC13A (Supplementary Table S1) in the TDP-43 dataset PRJEB42763 (Brown *et al.* 2022). Since an important aspect of RNA-seq data research is the visualization of alignments, coverage, junctions, and genome annotation, *splicekit* provides a local JBrowse2 URL link for every reported genomic feature result. Opening the link visualizes all related sample data in the local web browser (Fig. 1C).

### 3.2 Comparing specific and non-specific splicing modifying drugs

To evaluate and investigate the impact of a treatment on splicing compared to overall gene expression, we perform *splicekit* analysis on a Branaplam dataset GSE221868 (Ishigami *et al.* 2024, Supplementary Table S2). Branaplam is a known small molecule splicing modifier and its resulting juDGE plot (Fig. 1D, score = 0.22) is narrower than the juDGE plot of other compounds, e.g. Sorafenib, a kinase inhibitor for cancer treatment (Supplementary Fig. S1, score = 0.55). This implies that Sorafenib has a greater influence on gene expression alterations than Branaplam. We propose using juDGE plots as a method to estimate the degree to which a treatment affects splicing changes versus gene expression changes.

### 3.3 Multi-level analysis of junction, exon, and gene expression

When examining multiple treatments simultaneously, another valuable preliminary analysis involves clustering treatments based on significant impacts at the junction, exon, and gene levels. We analyze the dataset of acute myeloid leukemia GSE182150 (Curtiss *et al.* 2022, Supplementary Table S3), in which samples are grouped by experimental factors (time/cell type) and further subdivided by treatment (Fig. 1E). This demonstrates that the diverse time/cell type effects provide a strong foundation, which is then further modified by the treatment.

### 3.4 Spotlight on RBPs

To gain a deeper understanding of the involvement of RBPs in the splicing regulation across various comparisons, we have developed and incorporated scanRBP into *splicekit*. Once regulated junctions are pinpointed and classified (Fig. 2A), scanRBP can calculate protein binding profiles across provided sequences or genomic regions (Fig. 2B). After computing 100K bootstraps, we can visualize and assess the significance of each RBP in the context of splice site selection (Fig. 2C). As an illustration, we perform the analysis on the TDP-43 dataset PRJEB42763 (Brown *et al.* 2022) to demonstrate that *splicekit* can identify a global repression trend of TDP-43 across the detected regulated donor and acceptor splice sites (Fig. 2C, Supplementary Fig. S2).

### 3.5 Spotlight on DonJuAn

We revisit the Branaplam dataset (Ishigami *et al.* 2024) to demonstrate the donor-junction-anchor (DonJuAn) analysis. To categorize donor splice sites into repressed, enhanced, and control groups, we initially examine regulated junctions and select the significant ones (FDR < 0.05). We then compute anchors (15 nt regions upstream of the donor splice site) and combine the junction and anchor logFC signals. This enables more effective categorization, as junction reads are limited, and we regain some power by also considering the anchors. Utilizing the DonJuAn method, we can robustly detect the Branaplam motif (AGAGTAA); however, when only considering junctions, the detected motif enrichment is less pronounced (Supplementary Fig. S3).

## 4 Discussion

We introduce *splicekit*, an integrative toolset for analysing short-read RNA-seq datasets in the context of alternative splicing regulation. By integrating diverse analysis tools and methods, including external tools such as edgeR, DREME, and rMATS, as well as novel tools such as juDGE, DonJuAn, and scanRBP, *splicekit* provides a multifaceted approach to splicing analysis.

One of the key strengths of *splicekit* is its ability to interconnect basic feature analysis with motif search and RNA–protein binding analysis, allowing for a more in-depth understanding of splicing regulation. Additionally, *splicekit* provides exploratory tools for studying the mode of action of splicing in the context of different treatments and compounds.

*splicekit* is freely available and can be run on a single computer or on a compute cluster, making it a versatile tool for researchers with varying computational resources. Overall, we believe that the scientific community may benefit from adopting, using, and further developing *splicekit*.

## Supplementary Material

vbae121_Supplementary_Data

## Data Availability

The source code for *splicekit* can be found at https://github.com/bedapub/splicekit, and the software is also packaged through PyPI at https://pypi.org/project/splicekit. The package, together with its dependencies, can be installed with the command “pip install splicekit.” The public datasets analyzed can be accessed using the following accession numbers: PRJEB42763, GSE221868, and GSE182150.
