# Supplementary material for: splicekit: an integrative toolkit for splicing analysis from short-read RNA-seq
Source: Bioinform Adv. 2024 Aug 17;4(1):vbae121. doi: 10.1093/bioadv/vbae121 (PMC11364168; doi:10.1093/bioadv/vbae121)
Supplement: vbae121_Supplementary_Data [file vbae121_supplementary_data.pdf]

splicekit: an integrative toolkit for splicing analysis from short-read RNA-seq

Gregor Rot*†, Arne Wehling†, Roland Schmucki, Nikolaos Berntenis, Jitao David Zhang, Martin Ebeling*

Roche Pharmaceutical Research and Early Development, Roche Innovation Center Basel, Basel, Switzerland

* Corresponding authors. Email: gregor.rot@gmail.com, martin.ebeling@roche.com
† Equal contribution

SUPPLEMENTARY MATERIAL

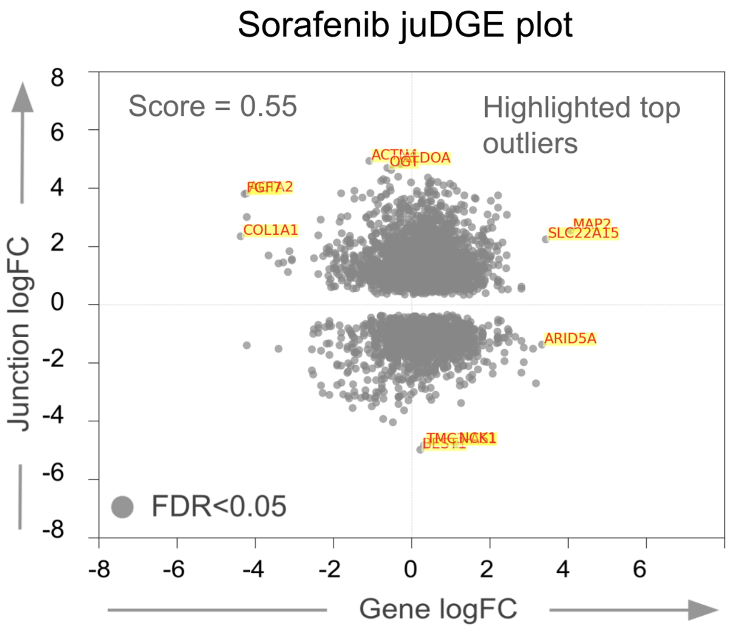

Figure S1
Sorafenib (dataset GSE98428) juDGE plot shows a wider profile (score = 0.55) compared to Branaplam, suggesting it has broader effects on differential gene expression.

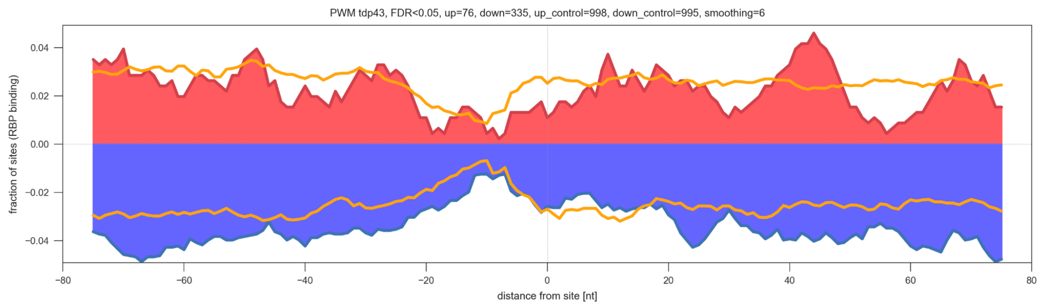

Figure S2

Predicted binding (PWM analysis on sequence) of TDP-43 at the acceptor regulated splice sites shows repression of the sites (p<1.00E-05, logFC = 0.44), however, to a lesser extent compared to donor sites (p<1.00E-05, logFC = 1.03). Acceptor splice sites that are up-regulated are not significantly bound (p=0.97, logFC=0.21), similar to up-regulated donor splice sites (p=0.64, logFC=0.04).

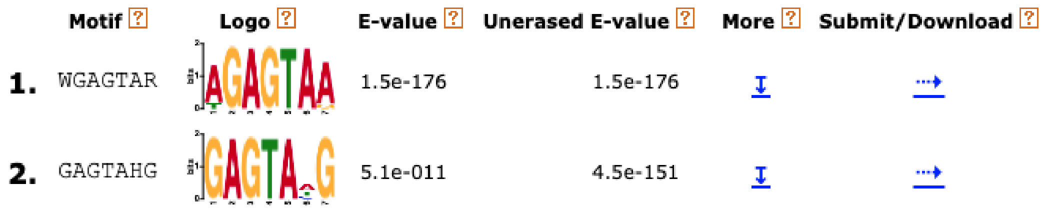

	Motif	Logo	E-value	Unerased E-value	More	Submit/Download
1.	WGAGTAR		1.5e-176	1.5e-176		
2.	GAGTAHG		5.1e-011	4.5e-151		

Figure S3

DREME motif analysis results for the Branaplam vs. control comparison (GSE221868) at the donor splice sites.

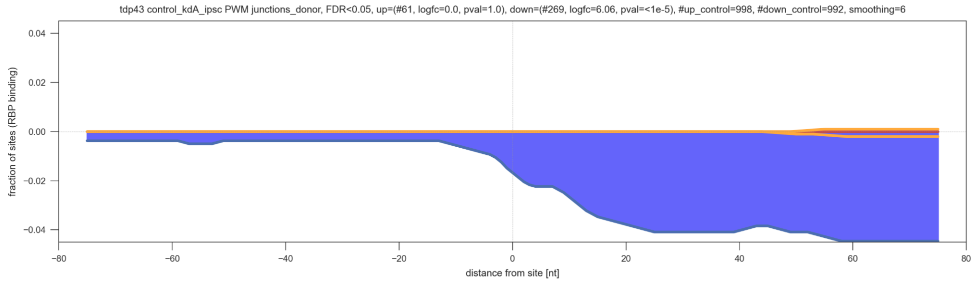

Figure S4

RNA-map of TDP-43 binding in K562 cells (eCLIP data, ENCODE project, ENCSR720BJU) at donor regulated sites in dataset PRJEB42763. We can note the majority of the signal is at downregulated donor sites in the intronic region, consistent with the predictive binding findings in Figure 2C.

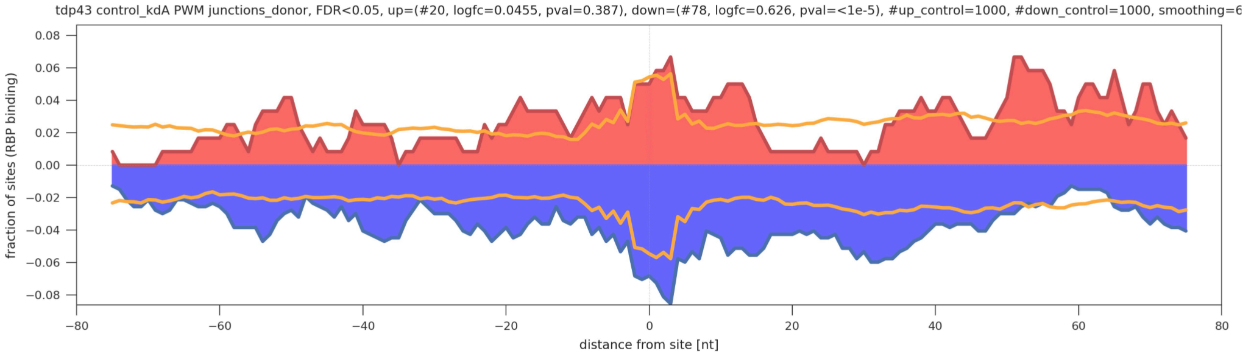

Figure S5
RNA-map of predicted TDP-43 binding (from mCross PWM) at donor regulated sites in mouse dataset PRJNA823722. Similarly to Figure 2C we see significant downregulation when binding is present.
This showcases the flexibility of splicekit also in terms of the choice of genome (via pybio) and moreover suggests that the scanRBP module (using PWMs) can be used on species different from the one on which PWMs available via scanRBP were derived (in this case human -> mouse).
We suggest caution when interpreting predictive results. An additional concern here is interpretation of signals from PWMs trained in one species (and context) applied to predict binding in another species (and context). In this case the similarity of the protein (cross-species) and the importance of the context must be considered when interpreting results.
In general, interpretation of predictive binding results (in this case from PWMs) is usually followed up with context specific experiments for validation.

Supplementary Tables

For detailed information on the table format, please refer to splicekit documentation online at https://github.com/bedapub/splicekit#filedescriptors.

Table S1
Junction analysis of dataset from Brown et al., PRJEB42763.

Table S2
Junction analysis of dataset from Ishigami et al., GSE221868.

Table S3
Junction analysis of dataset from Curtiss et al., GSE182150.
